# Investigations of Structural, Electronic and Magnetic Properties of MnSe under High Pressure

**DOI:** 10.3390/ma15031109

**Published:** 2022-01-31

**Authors:** Jing Zhao, Hanxing Zhang, Caoping Niu, Xianlong Wang

**Affiliations:** 1 Key Laboratory of Materials Physics, Institute of Solid State Physics, HFIPS, Chinese Academy of Sciences, Hefei 230031, China; jzhao@theory.issp.ac.cn (J.Z.); hxzhang@theory.issp.ac.cn (H.Z.); cpniu@theory.issp.ac.cn (C.N.); 2 Science Island Branch of Graduate School, University of Science and Technology of China, Hefei 230026, China

**Keywords:** high pressure, first-principles method, phase transition, spin crossover, MnSe

## Abstract

Properties of pressurized MnSe were investigated based on the first-principles methods using exchange–correlation functionals of the local density approximation (generalized gradient approximation) with and without the Hubbard U correction. Our results show that the Hubbard U (U = 4 eV) correction is necessary to correctly describe the phase transition behaviors of MnSe. We found that at the static condition, phase transitions from the low-temperature phase with a NiAs-type structure (P6_3_/mmc) to the P4/nmm phase at 50.5 GPa and further to the Pnma phase at 81 GPa are observed. However, if the transition starts from the room-temperature phase with a NaCl-type structure (Fm-3m), the transition-sequences and -pressures will be different, indicating that temperature can strongly affect the phase transition behaviors of MnSe. Furthermore, we found that pressure-induced negative charge transfer will promote spin crossover. The calculated superconducting properties of the Pnma phase indicate that it may be an unconventional superconductor.

## 1. Introduction

Transition metal chalcogenides have aroused great interest due to their rich optical, magnetic and transport properties [1,2,3,4,5,6] and have wide potential applications in infrared detectors, solar cells and spintronics devices [7,8]. Furthermore, high-pressure properties of manganese chalcogenides, such as MnSe and MnS_2_, have also attracted attention, since they exhibit large cell volume collapse and spin crossover during pressure-induced phase transitions [9,10,11]. Interestingly, a recent study reported the observation of superconductivity with T_C_ ~ 9 K in pressurized MnSe at ~35 GPa [12]. However, the superconducting structure is not clear yet.

Actually, controversies still exist in the reported phase transition behaviors of MnSe. Low-temperature neutron diffraction studies have shown that MnSe will undergo a transformation from a NaCl-type cubic structure with Fm-3m symmetry to a NiAs-type hexagonal structure with P6_3_/mmc symmetry when the temperature is lower than 266 K [13]. However, the same transition was observed at 140 K during the cooling process performed by means of synchrotron X-ray and neutron diffraction [14]. Furthermore, experimental works based on the in situ synchrotron XRD pattern [12] and the in situ angle dispersive synchrotron X-ray diffraction pattern [9] demonstrated that at room temperature, the low-pressure (LP) (0~12 GPa) and high-pressure (HP) (>30 GPa) phase structures of MnSe are NaCl-type with Fm-3m symmetry and MnP-type with Pnma symmetry, respectively. However, in [9], it is pointed out that there is an unknown tetrahedral structure between the Fm-3m and Pnma phases. The study also indicates the coexistence of the Fm-3m and P6_3_/mmc phases between 12.2 GPa and 16 GPa and the coexistence of the Fm-3m, P6_3_/mmc and Pnma phases between 16 GPa and 30 GPa [12]. On the other hand, the existing theoretical works [15,16] mainly studied the electronic structures of the Fm-3m phase, and there is a lack of reports on the structural evolution of MnSe under high pressure. We found that although lots of works had been conducted to explore the structures of MnSe, the evolution of the structure with pressure has not been clarified yet, especially the mixed phases between the Fm-3m phase and the Pnma phase observed by the experiment [9,12].

Furthermore, theoretical [10] and experimental [9,12] results suggested that the spin state transition, metal–insulator transition and superconductivity can be found in pressurized MnSe, but the superconducting mechanism has not yet been elucidated. Studying the evolution of magnetic moments and structures with pressure can give assistance to gain further insight into the mechanism of superconductivity in MnSe. Therefore, it is necessary to conduct a structure search for clarifying the phase transition behaviors of pressurized MnSe. In addition, the high-pressure phase transition behaviors of MnSe can provide a reference for the high-pressure phase transition behaviors of other manganese chalcogenides. For example, an experimental result measured in situ in a diamond anvil cell (DAC) apparatus found that MnS nanorods also have mixed phases in the pressure range of 16–25 GPa [17].

Based on the first-principles method combined with the structure-searching method, the properties of MnSe under high pressure are systematically investigated. We found that the structural phase transition sequences of MnSe are sensitive to the starting phase (or temperature). The behaviors of metal–insulator transition and spin crossover are illustrated, and pressure-induced negative charge transfer can promote spin crossover. The superconducting properties of the high-pressure phase are also presented.

## 2. Methods

The density functional theory (DFT) calculations were performed with the projector-augmented plane wave (PAW) potentials [18,19], as implemented in the Vienna Ab initio Simulation Package (VASP) [20]. There are two reasons for us to choose PAW potentials: (1) in the VASP package, PAW potentials are among the standard potentials, which are commonly used; (2) in our previous works [6,21], PAW pseudopotentials in the VASP package were successfully used to simulate the properties of Mn-based compounds. At first, the structures were fully relaxed based on the PAW potentials, and then the related properties were investigated, also based on the PAW potentials. The energy cutoff for the plane wave expansion was 360 eV. The convergence values of energy and force were set to 1 × 10^−6^ eV and 0.001 eV/Å, respectively. The first Brillouin zone was represented by the Monkhorst–Pack scheme [22,23], and the number of k points was sufficiently dense (k × a = 40, k × b = 40, and k × c = 40) to bring about convergent results. The exchange and correlation energy were described within local density approximation [24] (LDA), generalized gradient approximation (GGA) parametrized using the Perdew–Burke–Ernzerhof functional [25], LDA with the Hubbard U correction (LDA + U) and GGA with the Hubbard U correction (GGA + U) [26]. The onsite Coulomb interaction U of 1 eV, 2 eV, 3 eV and 4 eV was used for the Mn-*d* electrons. All the studied structures were completely relaxed until the total stress tensor was reduced to 0.01 GPa. Because it was experimentally reported that the magnetic moment of NaCl-type MnSe with Fm-3m symmetry was along the [111] direction [14,27], we used GGA and GGA + U (U = 1 eV, 2 eV, 3 eV, and 4 eV) based on Dudarev’s method [26] to simulate the energy of configuration of the magnetic moment along the [001] and [111] directions, respectively. The orientations of the magnetic moment of all the structures were set using the spin quantization axis (SAXIS-tag) in the VASP code. All the magnetic moments were given with respect to the axis, where all the magnetic moments written or read by VASP were given with respect to this axis. For example, when the direction of the magnetic moment is along [001], the quantization axis is set to “SAXIS = 0 0 1”. The results showed that the energy of the system for a magnetic moment along the [111] direction was only 0.25 meV/atom lower than that along the [001] direction, indicating that the energy was not very sensitive to the direction of the magnetic moment. Therefore, the magnetic moment in all the calculations was set along the [001] direction. Furthermore, the enthalpies of formation (H) of all the structures were calculated as follows:

(1) H=E+PV

where E, P and V are the static energies of structure, pressure on the structure and the volume of a system in equilibrium at 0 K, respectively. The calculations of ferromagnetic (FM) and antiferromagnetic (AFM) states depend on the parameters (ISPIN-tag and MAGMOM-tag) in the VASP program, and the spin polarization switch must be turned on during the calculation. For ISPIN = 1, non-spin-polarized calculations are performed, whereas for ISPIN = 2, spin-polarized calculations are performed. In addition, we also need to use MAGMOM-tag to specify the size of the magnetic moment. Taking MnSe whose unit cell contains two Mn atoms and two Se atoms as an example, the parameters for calculating the AFM state with an initial magnetic moment of 5 *μ_B_* should be set as “ISPIN = 2” and “MAGMOM = 5 −5 0 0” and the corresponding FM state should be set as “ISPIN = 2” and “MAGMOM = 5 5 0 0”. The phonon dispersions of all the structures were calculated using the density functional perturbation theory (DFPT) method within the LDA + U (U = 4 eV) functional, as implemented in the PHONOPY code [28,29]. Crystal structure searching at 0, 20, 80 and 100 GPa was carried out by using the Crystal structure AnaLYsis by Particle Swarm Optimization (CALYPSO) code [30,31].

Calculation of electron–phonon coupling (EPC) is not available in the VASP package (version 5.4.4), so we used the Quantum Espresso (QE) [32] package to simulate the superconducting properties of the structure, such as the electron–phonon coupling constant and the superconducting transition temperature. Therefore, we performed EPC calculations using the QE code with the DFPT method based on the Bardeen–Cooper–Schrieffer (BCS) theory [33]. The selection of pseudopotentials in the QE package is more flexible, so it is necessary to choose the type of pseudopotentials carefully. According to [34], the best pseudopotentials for simulating the properties of Mn and Se using the QE package are ultrasoft pseudopotentials (USPP) [35]. Therefore, the interactions between electrons and the ion core were described using USPP in this work. All the structures were fully relaxed until the Hellmann–Feynman force acting on each atom was less than 10^−5^ Ry/Å, and the convergence criterion for self-consistent calculations was set to 10^−6^ Ry. The energy cutoff of the wave functions and charge density were set to 80 Ry and 640 Ry, respectively, and 4 × 6 × 4 k-point meshes were used, both of which ensure the convergence of energies within 0.0001 Ry/atom. The related calculations of the superconducting properties were performed based on the fully relaxed structure, and we used 2 × 1 × 2 q-point meshes for EPC parameter λ, which represents the strength of the electron–phonon interaction. The larger the EPC constant λ, the higher the superconducting transition temperature of conventional superconductors.

## 3. Results and Discussion

### 3.1. Structural Evolution under Pressure

**Figure 1:**
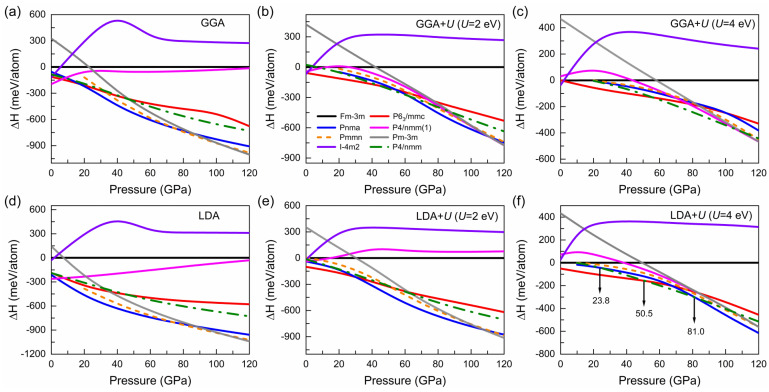
Enthalpies difference (ΔH) using generalized gradient approximation (GGA), GGA with the Hubbard U correction (GGA + U) (U = 2 eV) and GGA + U (U = 4 eV) are shown in (**a**–**c**), respectively, as a function of pressure. Counterparts using local density approximation (LDA), LDA + U (U = 2 eV) and LDA + U (U = 4 eV) are shown in (**d**–**f**), respectively.

To study the properties of MnSe under high pressure and the pressure-driven phase transitions, we considered a NaCl-type structure with Fm-3m symmetry, a NiAs-type structure with P6_3_/mmc symmetry and a MnP-type structure with Pnma symmetry reported in previous studies [9,12,13]. Besides, we also used the CALYPSO software to search for structures based on the GGA and GGA + U functionals. The results based on the GGA functional show that the stable structures are the tetragonal phase with P4/nmm (1) symmetry at 0 GPa, the hexagonal phase with P6_3_/mmc symmetry at 20 GPa and the orthogonal phase with Pmmn symmetry at 80 GPa and 100 GPa, respectively. However, the structure search using the GGA + U (U = 4 eV) functional show that the stable structures at 0 GPa, 20 GPa, 80 GPa and 100 GPa are the tetragonal phase with I-4m2 symmetry, the hexagonal phase with P6_3_/mmc symmetry, the tetragonal phase with P4/nmm symmetry and the cubic phase with Pm-3m symmetry, respectively. The applied U value had strong effects on the searched stable structures. In combination with the structure search in the GGA and GGA + U framework, eight kinetic stable structures were selected to calculate the dependence of enthalpy on pressure (shown in Figure 1) through the GGA (LDA) and GGA (LDA) + U functionals.

As a function of pressure, Figure 1a–c show the evolution of the relative enthalpies by taking the Fm-3m phase as reference using the GGA and GGA + U (U = 2 eV and 4 eV) functionals. Obviously, correlation interaction (the choice of different U values) plays a crucial role in the description of phase transition sequences, leading to different stable HP phases in Figure 1a–c. Note that at 0 GPa, the most and the second most stable phases simulated by means of GGA and GGA + U are not the experimentally observed two stable phases at ambient pressure, a NaCl-type cubic structure with Fm-3m symmetry at room temperature and a NiAs-type hexagonal structure with P6_3_/mmc symmetry at lower temperature [13,14]. In addition, the results calculated using GGA + U (U = 1 eV and 3 eV) also cannot correctly describe the ground state structure of MnSe. Compared with GGA which usually overestimates the lattice constant, LDA slightly underestimates them [36]. A natural follow-up work is to look into the structural changes of MnSe under high pressure based on the LDA and LDA + U functionals. The results show that U = 4 eV can well describe the structure of MnSe when the U values of 1 eV, 2 eV, 3 eV, and 4 eV are applied. For ease of discussion, only the evolutions of relative enthalpies with pressure for U = 2 eV and U = 4 eV were plotted, respectively, as shown in Figure 1e–f. It can be found that compared to LDA and LDA + U (U = 2 eV), adopted U = 4 eV for the LDA + U scheme can accurately describe the stable MnSe phase at 0 GPa. The most and the second most stable phases present at 0 GPa (shown in Figure 1f) are the P6_3_/mmc phase and the Fm-3m phase, respectively, consistent with the experimental observations [13,14]. Therefore, in the following, the discussions about the HP properties are based on the results calculated using LDA + U (U = 4 eV), as shown in Figure 1f.

**Figure 2:**
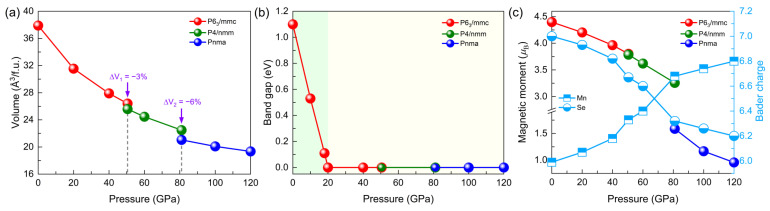
Evolution of (**a**) volume and (**b**) band gap based on the LDA + U (U = 4 eV) functional are shown as a function of pressure. In (**c**), magnetic moments (black, left Y-axis) and Bader charge (blue, right Y-axis) are presented. In (**a**), the vertical gray dashed lines indicate the pressure positions of the two-phase transitions in turn. In (**b**), green and yellow areas represent the stable ranges of the insulator and the metal, respectively.

According to Figure 1f, compared with the experimentally reported stable structure (NaCl-type cubic structure with Fm-3m symmetry at room temperature) at ambient pressure, a NiAs-type hexagonal structure with P6_3_/mmc symmetry at lower temperature has lower enthalpy, indicating that temperature affects the stability of the structure. We further compared the difference in Helmholtz free energy of the two structures at different temperatures. The results showed that the energy of the P6_3_/mmc phase was 50 meV/atom lower and 2 meV/atom higher than that of the Fm-3m phase at 0 K and 900 K, respectively, which suggests that the Fm-3m phase is more stable at high temperatures and the energy difference between the Fm-3m and P6_3_/mmc phases decreases with increasing temperature. As pressure increased, a phase transition from the P6_3_/mmc phase to the tetragonal phase with P4/nmm symmetry occurred, which involved a volume compression of 3%, evidencing the first-order phase transition (shown in Figure 2a). The P4/nmm phase had the lowest energy at the pressure range of 50.5 GPa~81.0 GPa, and it transformed into a MnP-type structure with Pnma symmetry combined with a volume reduction of 6% (Figure 2a). When the pressure was less than 10 GPa, two high-pressure phases, P4/nmm and Pnma phases, spontaneously transformed into Fm-3m and P6_3_/mmc structures, respectively. The above discussions suggest that the sequences of phase transition we simulated (P6_3_/mmc → P4/nmm → Pnma) at 0 K are slightly different from the experimentally observed results (Fm-3m → an unknown tetragonal phase → Pnma) [9] at room temperature, which is caused by the different stable structure of MnSe at room temperature and lower temperature. Please note that at 10 GPa, the enthalpies of the P4/nmm and Pnma phases are very close to the high-temperature phase at ambient pressure (Fm-3m phase). Therefore, if the Fm-3m phase is used for pressure loading, the phase transition to the Pnma phase and further to the P4/nmm phase may occur at ~10 GPa and 23.8 GPa, respectively. We found that temperature has strong effects not only on the stable phase at ambient pressure, but also on the high-pressure phase transition sequences, which may give rise to the coexistence of mixed phases at a large pressure range [9,12]. Please note, the tetragonal phase with P4/nmm symmetry has not been reported before and can provide a reference for illustrating the experimentally observed unknown tetrahedral structure between the Fm-3m and Pnma phases [9]. Based on the above discussion, we concluded that temperature modulates the phase transition sequences under pressure by affecting the stable structure under ambient pressure.

**Figure 3:**
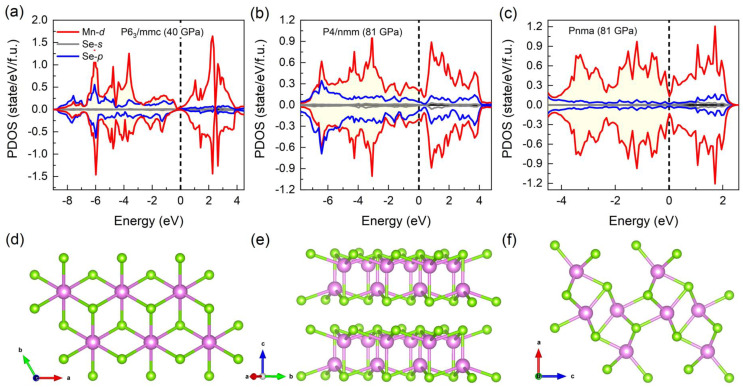
(**a**) Partial density of states (PDOS) of MnSe with P6_3_/mmc symmetry at 40 GPa. (**b**) PDOS of MnSe with P4/nmm symmetry at 81 GPa. (**c**) PDOS of MnSe with Pnma symmetry at 81 GPa. The ground-state structures of the P6_3_/mmc phase (**d**), the P4/nmm phase (**e**) and the Pnma phase (**f**), in which the Mn and Se atoms are represented by pink and green spheres, respectively. In (**a**–**c**), the dashed vertical lines show the Fermi energy level, and the yellow shadow area represents the occupied state of Mn-*d* electrons.

**Figure 4:**
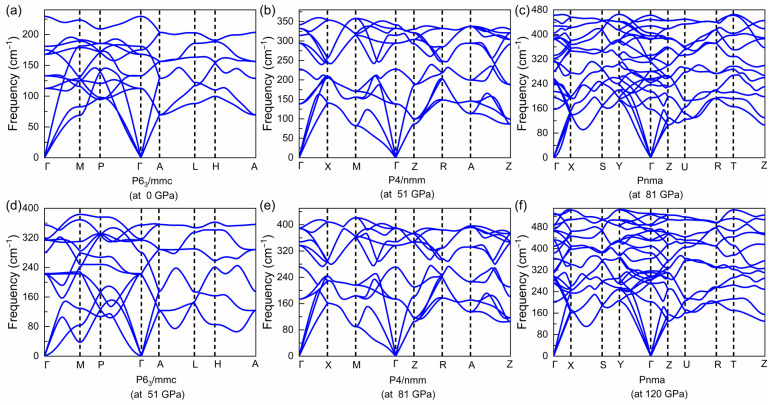
Phonon dispersions calculated using the LDA + U (U = 4 eV) functional with (**a**) P6_3_/mmc symmetry at 0 GPa, with (**b**) P4/nmm symmetry at 51 GPa, with (**c**) Pnma symmetry at 81 GPa, with (**d**) P6_3_/mmc phase at 51 GPa, with (**e**) P4/nmm phase at 81 GPa and with (**f**) Pnma phase at 120 GPa.

The corresponding structural details are summarized in Table 1, and the geometrical structures are shown in Figure 3d–f. In order to verify whether the three structures are still stable under different pressures, the phonon dispersion relationships under high pressure were calculated. No negative frequencies were observed in the phonon dispersions shown in Figure 4, which indicates that the three phases are dynamically stable. Furthermore, the second-order elastic constants shown in Table 2 meet the Born stability criteria of the hexagonal (P6_3_/mmc) class as $C_{11}>\left|C_{12}\right|,\;2C_{13}^{2}<C_{33}\left(C_{11}+C_{12}\right),\;C_{44}>0,\;C_{66}>0$, of the tetragonal (P4/nmm) class as $C_{11}>\left|C_{12}\right|,\;2C_{13}^{2}<C_{33}\left(C_{11}+C_{12}\right),\;C_{44}>0,\;C_{66}>0$ and of the orthorhombic (Pnma) phase as $C_{11}>0,\;C_{44}>0,\;C_{55}>0,\;C_{66}>0,\;C_{11}C_{22}>C_{12}^{2},\;C_{11}C_{22}C_{33}+2C_{12}C_{13}C_{23}-C_{11}C_{23}^{2}-C_{22}C_{13}^{2}-C_{33}C_{12}^{2}>0$ [37]. These results indicate the three phases are kinetically and mechanically stable.

### 3.2. Electronic and Magnetic Properties

**Figure 5:**
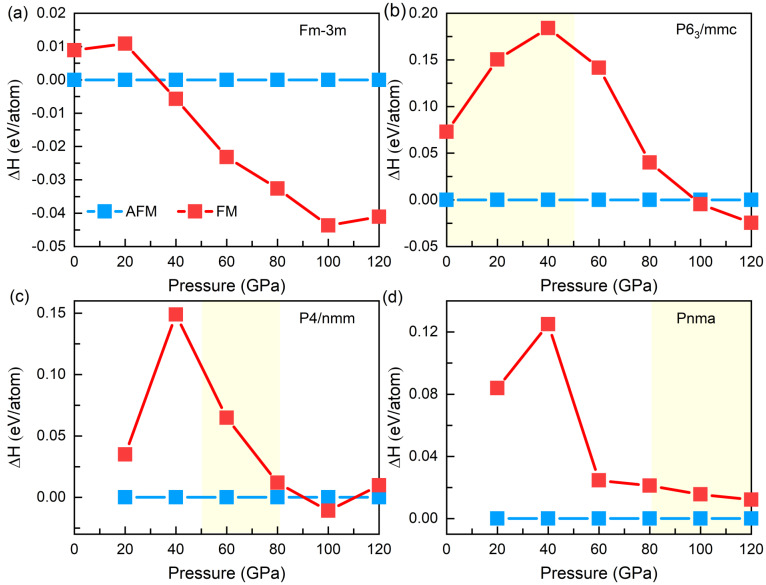
Evolution of enthalpy difference (ΔH) between the anti-ferromagnetism (AFM) and ferromagnetism (FM) of (**a**) the Fm-3m phase, (**b**) the P6_3_/mmc phase, (**c**) the P4/nmm phase and (**d**) the Pnma phase. In (**b**–**d**), the yellow region indicates the stable range of MnSe with P6_3_/mmc symmetry, with P4/nmm symmetry and with Pnma symmetry, respectively.

In this section, we discuss the evolution of magnetic states of these stable structures with Fm-3m symmetry, P6_3_/mmc symmetry, P4/nmm symmetry and Pnma symmetry. The energy of the non-magnetism (NM) state was much higher than that of the FM and AFM states within the investigated pressure range (0~120 GPa). Figure 5 represents the calculated pressure dependence of enthalpy difference between the AFM and FM states. Calculations based on LDA + U (U = 4 eV) provided an AFM state with a 4.51 *μ_B_* magnetic moment, the ground magnetic state of the Fm-3m phase at 0 GPa, which is consistent with theoretical [16] and experimental results [38]. Except the Pnma phase, pressure can induce a magnetic transition from AFM to FM in the other three phases. However, AFM is always the ground magnetic state in their thermodynamically stable pressure range, which means that the AFM state has the lowest enthalpy value compared to FM in the corresponding pressure range. For example, as shown in Figure 1f and Figure 5b, the P6_3_/mmc phase with AFM state has the lowest enthalpy in the pressure range of 0–50.5 GPa.

Furthermore, the band gaps of the P6_3_/mmc phase, the P4/nmm phase and the Pnma phase are shown as a function of pressure in Figure 2b. The band gap of the P6_3_/mmc phase gradually decreases to 0 eV at 20 GPa, leading to a metal–insulator transition, comparable to the results reported in the experiment [9]. The analysis of the partial density of states (PDOS) (Figure 3a–c) of the P6_3_/mmc phase at 40 GPa, the P4/nmm phase at 81 GPa and the Pnma phase at 81 GPa show that metallization originates from the itinerant Mn-*d* electrons as well as the increased density of states at the Fermi level. Evolutions of the magnetic moment and Bader charge with pressure are shown in Figure 2c; we found that the magnetic moment of MnSe decreased with pressure increasing. The magnetic moment gradually dropped from a high-spin state (4.40 *μ_B_*) at 0 GPa to 3.62 *μ_B_* at 60 GPa and then rapidly dropped to 1.59 *μ_B_* at 81 GPa. Finally, the low-spin state with a magnetic moment of 0.96 *μ_B_* was achieved at the pressure of 120 GPa. The results indicate that pressure-driven spin crossover of MnSe occurs at a large pressure range (0~120 GPa). Furthermore, with pressure increasing, negative charge transfer from Se to Mn can be found, as shown in Figure 2c, and the decreasing (increasing) behavior of the Bader charge in Se (Mn) atoms as a function of pressure is similar to the counterpart of Mn’s magnetic moment. The results mean that the negative charge transfer promotes the spin crossover of MnSe.

### 3.3. Superconducting Properties of the Pnma Phase

**Figure 6:**
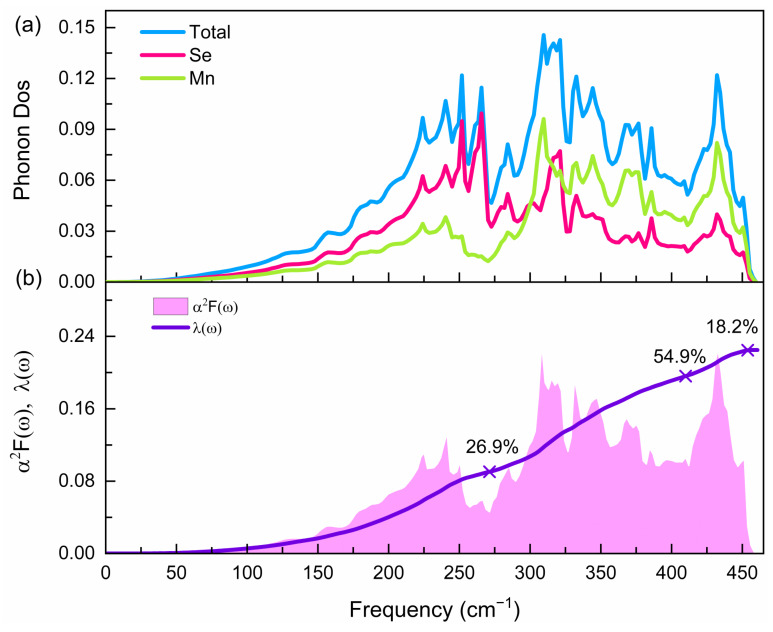
(**a**) Partial phonon density of states (Phonon Dos) of the Pnma phase at 81 GPa. (**b**) Eliashberg phonon spectral function $\alpha^{2}F\left(\omega \right)$ and electron—phonon coupling (EPC) parameter λ.

Finally, we show the superconducting properties and phonon density of states of the Pnma phase at 81 GPa, as shown in Figure 6a. The total phonon density can be divided into three parts. The low frequency region within 271 cm^−1^ mainly comes from the vibration of Se atoms. The intermediate frequency region within 271~413 cm^−1^ and the high frequency region above 413 cm^−1^ mainly come from the vibration of Mn atoms. The calculated Eliashberg phonon spectral function $\alpha^{2}F\left(\omega \right)$ and EPC parameter λ representing the strength of the electron–phonon interaction are plotted in Figure 6b. The total EPC strength is as follows:

(2) $$\lambda \left(\omega \right)=2\int_{0}^{\omega }\frac{\alpha^{2}F\left(\omega \right)}{\omega }d\omega$$

EPC with a smaller value is distributed across the phonon mode range. The contributions of low-frequency (below 271 cm^−1^), medium-frequency (271~413 cm^−1^), as well as high-frequency (above 413 cm^−1^) vibration accounted for 26.9%, 54.9% and 18.2% of the total EPC constant, respectively. Based on the Allen–Dynes equation [39,40], the calculated superconducting transition temperature is very close to 0 K (0.004 K), which is far smaller than the experimentally reported value [12]. The smaller superconducting transition temperature indicates that MnSe may not be a conventional superconductor.

## 4. Conclusions

In this work, the high-pressure properties, especially the high-pressure phase structures, of MnSe were systematically investigated based on the first-principles methods combined with the structure-searching method. The Hubbard U correction is necessary for describing the phase stability of MnSe correctly, and a new tetragonal phase with P4/nmm symmetry was predicted to clarify the phase transition sequence of pressurized MnSe. The phase transition behaviors of MnSe notably depend on temperature or starting phases, which may result in the existence of mixed phases in a large pressure range. Pressure-induced negative charge transfer can promote spin crossover, and MnSe with Pnma symmetry may be an unconventional superconductor.

## Figures and Tables

**Table 1:** The calculated crystal structure parameters (space group (SG), number of formula units in unit cell Z, lattice parameters (*a*, *b* and *c*), Wyckoff site and the corresponding coordinates (*x*, *y* and *z*)) of MnSe with P6_3_/mmc symmetry at 0 GPa, with P4/nmm symmetry at 60 GPa and with Pnma symmetry at 100 GPa.

P (GPa)	SG	Z	*a* (Å)	*b* (Å)	*c* (Å)	WP	*x*	*y*	*z*
0	P6_3_/mmc	2	3.761	3.761	6.188	Mn(2a)	0.0000	0.0000	0.0000
						Se(2c)	0.3333	0.6667	0.2500
60	P4/nmm	2	3.160	3.160	4.898	Mn(2c)	0.0000	0.5000	0.1553
						Se(2c)	0.5000	−0.000	0.3134
100	Pnma	4	5.265	2.891	5.280	Mn(4c)	−0.015	0.7500	0.3065
						Se(4c)	−0.197	0.7500	0.9281

**Table 2:** C_ij_ elastic constants (in GPa) of MnSe with the P6_3_/mmc phase at 0 GPa, with the P4/nmm phase at 50.5 GPa and with the Pnma phase at 81 GPa.

Space Group	P6_3_/mmc	P4/nmm	Pnma
	(0 GPa)	(50.5 GPa)	(81 GPa)
C_11_	122.8	178.0	361.7
C_12_	54.1	97.9	261.2
C_13_	49.7	89.3	180.3
C_22_	122.8	178.0	426.6
C_23_	49.7	89.3	200.1
C_33_	142.4	385.5	449.1
C_44_	36.9	72.8	171.5
C_55_	36.9	72.8	234.7
C_66_	34.4	169.4	271.5

## Data Availability

Not applicable.
